# 1-Chloro­acetyl-2,6-bis­(2-chloro­phen­yl)-3,5-dimethyl­piperidin-4-one oxime

**DOI:** 10.1107/S1600536810018489

**Published:** 2010-05-29

**Authors:** K. Ravichandran, P. Ramesh, M. Rani, S. Kabilan, M. N. Ponnuswamy

**Affiliations:** a Centre of Advanced Study in Crystallography and Biophysics, University of Madras, Guindy Campus, Chennai 600 025, India; b Department of Chemistry, Annamalai University, Annamalainagar 608 002, Tamil Nadu, India

## Abstract

In the title compound, C_21_H_21_Cl_3_N_2_O_2_, the piperidine ring adopts a distorted boat conformation. One of the chloro­phenyl rings is almost perpendicular to the best plane through piperidine ring, making a dihedral angle of 88.7 (1)°, whereas the other ring is twisted by 71.8 (1)°. The crystal packing is stabilized by inter­molecular C—H⋯O, C—H⋯Cl and O—H⋯O inter­actions.

## Related literature

For general background to piperidine derivatives, see: Perumal *et al.* (2001 ▶); Dimmock *et al.* (2001 ▶); Ravindran *et al.* (1991 ▶); Senthilkumar *et al.* (1992 ▶). For the synthesis of the title compound, see: Aridoss *et al.* (2007 ▶). For asymmetry parameters, see: Nardelli (1983 ▶). For puckering parameters, see: Cremer & Pople (1975 ▶). For hydrogen-bond motifs, see: Bernstein *et al.* (1995 ▶).
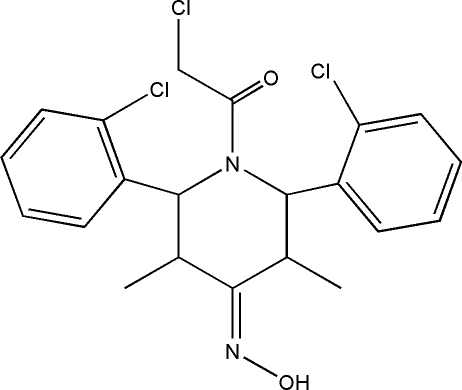


## Experimental

### 

#### Crystal data

C_21_H_21_Cl_3_N_2_O_2_
*M* *_r_* = 439.75
Monoclinic, 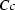

*a* = 9.8147 (6) Å
*b* = 15.5929 (11) Å
*c* = 13.9498 (9) Å
β = 93.529 (4)°
*V* = 2130.8 (2) Å^3^
*Z* = 4
Mo *K*α radiation
μ = 0.45 mm^−1^
*T* = 293 K
0.23 × 0.19 × 0.17 mm

#### Data collection

Bruker SMART APEXII area-detector diffractometer
Absorption correction: multi-scan (*SADABS*; Bruker, 2008 ▶) *T*_min_ = 0.903, *T*_max_ = 0.927
10047 measured reflections
4689 independent reflections
4181 reflections with *I* > 2σ(*I*)
*R*_int_ = 0.019

#### Refinement

*R*[*F* ^2^ > 2σ(*F* ^2^)] = 0.031
*wR*(*F* ^2^) = 0.076
*S* = 1.03
4689 reflections
255 parameters
2 restraints
H-atom parameters constrained
Δρ_max_ = 0.20 e Å^−3^
Δρ_min_ = −0.18 e Å^−3^
Absolute structure: Flack (1983 ▶), 2045 Friedel pairs
Flack parameter: 0.04 (4)

### 

Data collection: *APEX2* (Bruker, 2008 ▶); cell refinement: *SAINT* (Bruker, 2008 ▶); data reduction: *SAINT*; program(s) used to solve structure: *SHELXS97* (Sheldrick, 2008 ▶); program(s) used to refine structure: *SHELXL97* (Sheldrick, 2008 ▶); molecular graphics: *ORTEP-3* (Farrugia, 1997 ▶); software used to prepare material for publication: *SHELXL97* and *PLATON* (Spek, 2009 ▶).

## Supplementary Material

Crystal structure: contains datablocks global, I. DOI: 10.1107/S1600536810018489/bt5245sup1.cif

Structure factors: contains datablocks I. DOI: 10.1107/S1600536810018489/bt5245Isup2.hkl

Additional supplementary materials:  crystallographic information; 3D view; checkCIF report

## Figures and Tables

**Table 1:** Hydrogen-bond geometry (Å, °)

*D*—H⋯*A*	*D*—H	H⋯*A*	*D*⋯*A*	*D*—H⋯*A*
O2—H2*A*⋯O1^i^	0.82	2.01	2.7515 (18)	150
C12—H12⋯Cl2^ii^	0.93	2.68	3.485 (3)	145
C20—H20⋯O1^iii^	0.93	2.42	3.158 (3)	136

Symmetry codes: (i) 
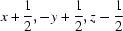
; (ii) 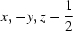; (iii) 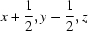.

**Fig. 1.**
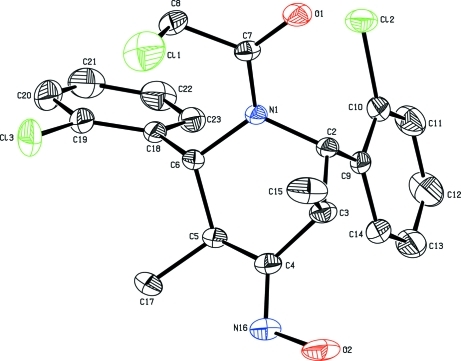
Perspective view of the molecule showing the thermal ellipsoids are drawn at 30% probability level. H atoms have been omitted for clarity.

**Fig. 2.**
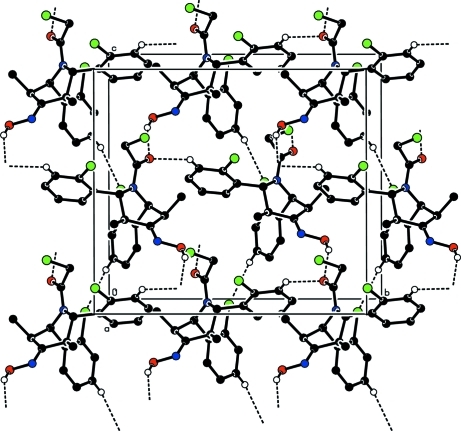
The crystal packing of the molecules viewed down a–axis. H atoms not involved in hydrogen bonding have been omitted for clarity.

## supplementary crystallographic information

### Comment

Piperidine derivatives are the valued heterocyclic compounds in the field of medicinal chemistry. Piperidin-4-ones are reported to possess analgesic, anti-inflammatory, central nervous system (CNS), local anaesthetic, anti-cancer and anti-microbial activities (Perumal *et al.* 2001; Dimmock *et al.*,  2001). However, introduction of certain heteroconjugate groups such as –NO, –CHO, –COCH3, –COC_6_H_5_, etc., at the ring nitrogen of 2,6-disubstituted piperidine ring system have reported to cause a major change in ring conformation, chemical shifts of carbons and associated protons in addition to the orientation of substituents (Ravindran *et al.*,  1991; Senthilkumar *et al.*,  1992). The crystallographic study of the title compound has been carried out to establish the molecular structure.

The piperidine ring in the molecule (Fig. 1) adopts a distorted boat conformation with the puckering parameters (Cremer & Pople, 1975) and the asymmetry parameters (Nardelli, 1983) are: q_2_ = 0.673 (2) Å, q_3_ = 0.080 (2) Å, φ_2_ = 70.2 (2)° and Δ_s_(C2 & C5)= 10.5 (2)°. One of the chlorophenyl rings is almost perpendicular to the best plane of piperidine ring with a dihedral angle of 88.7 (1)°, whereas the other ring is twisted by 71.8 (1)°. The sum of the bond angles around the atom N1 (357.4°) of the piperidine ring in the molecule is in accordance with sp2 hybridized state. The chloro-acetyl group adpots a twist conformation which can be seen from the torsion angle of 91.1 (2)° [N1—C7—C8—Cl1].

The crystal packing is controlled by C—H···O, C—H···Cl and O—H···O  types of intermolecular interactions, which form a three dimensional network. Atoms O2 and C20 of the molecule at (x, y, z) donate a proton to bifurcated acceptor atom O1 of the molecule at (1/2+x, 1/2-y, -1/2+z & 1/2+x, -1/2+y, z), forming two different C(9) and C(8) chains (Bernstein *et al.*, 1995) running along the ac diagonal and b-axis, respectively, as shown in Fig. 2.

### Experimental

A mixture of *N*-chloroacetyl-3,5-dimethyl-2,6-bis(*o*-chlorophenyl piperridin-4-one1 (50 mmol), sodiumacetate trihydrate (150 mmol), hydroxylamine hydrochloride (60 mmol) and 50 ml of ethanol were taken in a RB flask. The reaction mixture was refluxed for about half an hour. The progress of the reaction was monitored by TLC. After the usual workup, the oxime was purified by column chromatography and the single crystals were grwon by slow evaporation using ethanol as solvent (Aridoss *et al.*, 2007).

### Refinement

H atoms were positioned geometrically (C—H = 0.93 - 0.98 Å, O—H = 0.82Å) and allowed to ride on their parent atoms, with 1.5U_eq_(C) for methyl, 1.5_eq_(O) for oxygen H and 1.2 U_eq_(C) for other H atoms.

### Crystal data

**Table:**

C_21_H_21_Cl_3_N_2_O_2_	*F*(000) = 912
*M**_r_* = 439.75	*D*_x_ = 1.371 Mg m^−^^3^
Monoclinic, *C**c*	Mo *K*α radiation, λ = 0.71073 Å
Hall symbol: C -2yc	Cell parameters from 1231 reflections
*a* = 9.8147 (6) Å	θ = 2.5–28.3°
*b* = 15.5929 (11) Å	µ = 0.45 mm^−^^1^
*c* = 13.9498 (9) Å	*T* = 293 K
β = 93.529 (4)°	Block, colorless
*V* = 2130.8 (2) Å^3^	0.23 × 0.19 × 0.17 mm
*Z* = 4	

### Data collection

**Table:**

Bruker SMART APEXII area-detector diffractometer	4689 independent reflections
Radiation source: fine-focus sealed tube	4181 reflections with *I* > 2σ(*I*)
graphite	*R*_int_ = 0.019
ω and φ scans	θ_max_ = 28.3°, θ_min_ = 2.5°
Absorption correction: multi-scan (*SADABS*; Bruker, 2008)	*h* = −13→13
*T*_min_ = 0.903, *T*_max_ = 0.927	*k* = −20→20
10047 measured reflections	*l* = −18→18

### Refinement

**Table:**

Refinement on *F*^2^	Secondary atom site location: difference Fourier map
Least-squares matrix: full	Hydrogen site location: inferred from neighbouring sites
*R*[*F*^2^ > 2σ(*F*^2^)] = 0.031	H-atom parameters constrained
*wR*(*F*^2^) = 0.076	*w* = 1/[σ^2^(*F*_o_^2^) + (0.0346*P*)^2^ + 0.6047*P*] where *P* = (*F*_o_^2^ + 2*F*_c_^2^)/3
*S* = 1.03	(Δ/σ)_max_ < 0.001
4689 reflections	Δρ_max_ = 0.20 e Å^−^^3^
255 parameters	Δρ_min_ = −0.18 e Å^−^^3^
2 restraints	Absolute structure: Flack (1983), 2045 Friedel pairs
Primary atom site location: structure-invariant direct methods	Flack parameter: 0.04 (4)

### Special details

**Table:**

Geometry. All esds (except the esd in the dihedral angle between two l.s. planes) are estimated using the full covariance matrix. The cell esds are taken into account individually in the estimation of esds in distances, angles and torsion angles; correlations between esds in cell parameters are only used when they are defined by crystal symmetry. An approximate (isotropic) treatment of cell esds is used for estimating esds involving l.s. planes.
Refinement. Refinement of *F*^2^ against ALL reflections. The weighted *R*-factor wR and goodness of fit *S* are based on *F*^2^, conventional *R*-factors *R* are based on *F*, with *F* set to zero for negative *F*^2^. The threshold expression of *F*^2^ > σ(*F*^2^) is used only for calculating *R*-factors(gt) etc. and is not relevant to the choice of reflections for refinement. *R*-factors based on *F*^2^ are statistically about twice as large as those based on *F*, and *R*- factors based on ALL data will be even larger.

### Fractional atomic coordinates and isotropic or equivalent isotropic displacement parameters (Å^2^)

**Table:**

	*x*	*y*	*z*	*U*_iso_*/*U*_eq_	
Cl1	0.88541 (8)	0.18026 (6)	0.71303 (5)	0.0929 (3)	
Cl2	0.40087 (5)	0.06564 (4)	0.47110 (4)	0.05560 (15)	
Cl3	1.07034 (7)	−0.00744 (6)	0.60948 (5)	0.0826 (2)	
O1	0.57421 (15)	0.18007 (9)	0.60868 (9)	0.0470 (3)	
O2	0.91903 (18)	0.31451 (10)	0.26625 (12)	0.0605 (4)	
H2A	0.9746	0.3323	0.2295	0.091*	
N1	0.71338 (13)	0.12798 (9)	0.49843 (9)	0.0296 (3)	
C2	0.64401 (17)	0.18068 (11)	0.42143 (12)	0.0328 (3)	
H2	0.5740	0.2141	0.4519	0.039*	
C3	0.74830 (19)	0.24562 (13)	0.38710 (13)	0.0400 (4)	
H3	0.7049	0.2779	0.3332	0.048*	
C4	0.87049 (17)	0.19941 (13)	0.35168 (12)	0.0372 (4)	
C5	0.89159 (17)	0.10640 (13)	0.37891 (12)	0.0365 (4)	
H5	0.8338	0.0725	0.3334	0.044*	
C6	0.84595 (16)	0.08546 (12)	0.48021 (11)	0.0326 (4)	
H6	0.9157	0.1072	0.5274	0.039*	
C7	0.67616 (17)	0.13972 (11)	0.58982 (12)	0.0334 (4)	
C8	0.7634 (2)	0.10242 (15)	0.67283 (13)	0.0480 (5)	
H8A	0.8093	0.0511	0.6523	0.058*	
H8B	0.7066	0.0870	0.7247	0.058*	
C9	0.56989 (17)	0.12926 (12)	0.34100 (11)	0.0343 (4)	
C10	0.45857 (18)	0.07723 (13)	0.35647 (13)	0.0403 (4)	
C11	0.3877 (2)	0.03212 (19)	0.28473 (16)	0.0584 (6)	
H11	0.3146	−0.0027	0.2987	0.070*	
C12	0.4266 (2)	0.0394 (2)	0.19198 (18)	0.0749 (8)	
H12	0.3798	0.0094	0.1427	0.090*	
C13	0.5331 (2)	0.0905 (2)	0.17247 (14)	0.0668 (7)	
H13	0.5585	0.0956	0.1096	0.080*	
C14	0.6050 (2)	0.13518 (15)	0.24530 (13)	0.0467 (5)	
H14	0.6779	0.1698	0.2303	0.056*	
C15	0.7918 (3)	0.30940 (15)	0.46641 (19)	0.0625 (6)	
H15A	0.8360	0.2792	0.5196	0.094*	
H15B	0.7127	0.3386	0.4873	0.094*	
H15C	0.8539	0.3504	0.4421	0.094*	
N16	0.95359 (17)	0.23024 (11)	0.29455 (11)	0.0465 (4)	
C17	1.0382 (2)	0.07602 (18)	0.36986 (17)	0.0626 (7)	
H17A	1.0663	0.0894	0.3069	0.094*	
H17B	1.0430	0.0152	0.3799	0.094*	
H17C	1.0974	0.1044	0.4172	0.094*	
C18	0.83630 (18)	−0.01119 (13)	0.49265 (12)	0.0383 (4)	
C19	0.9322 (2)	−0.05775 (16)	0.54924 (15)	0.0545 (6)	
C20	0.9212 (3)	−0.14576 (19)	0.5596 (2)	0.0722 (8)	
H20	0.9846	−0.1754	0.5993	0.087*	
C21	0.8168 (3)	−0.18886 (17)	0.5115 (2)	0.0773 (9)	
H21	0.8093	−0.2479	0.5186	0.093*	
C22	0.7226 (3)	−0.14545 (15)	0.4525 (2)	0.0633 (6)	
H22	0.6528	−0.1751	0.4187	0.076*	
C23	0.7326 (2)	−0.05725 (13)	0.44401 (14)	0.0442 (4)	
H23	0.6682	−0.0281	0.4047	0.053*	

### Atomic displacement parameters (Å^2^)

**Table:**

	*U*^11^	*U*^22^	*U*^33^	*U*^12^	*U*^13^	*U*^23^
Cl1	0.0956 (5)	0.1161 (7)	0.0641 (4)	−0.0307 (5)	−0.0182 (3)	−0.0161 (4)
Cl2	0.0480 (2)	0.0744 (4)	0.0455 (2)	−0.0132 (3)	0.01178 (18)	0.0075 (3)
Cl3	0.0570 (3)	0.1203 (6)	0.0682 (4)	0.0274 (4)	−0.0150 (3)	0.0165 (4)
O1	0.0603 (8)	0.0469 (8)	0.0361 (6)	0.0160 (7)	0.0201 (6)	−0.0008 (6)
O2	0.0812 (10)	0.0457 (9)	0.0588 (9)	−0.0093 (8)	0.0375 (8)	0.0098 (7)
N1	0.0321 (6)	0.0311 (7)	0.0265 (6)	0.0048 (6)	0.0077 (5)	0.0017 (6)
C2	0.0351 (7)	0.0332 (9)	0.0312 (7)	0.0075 (7)	0.0108 (6)	0.0053 (8)
C3	0.0492 (9)	0.0333 (9)	0.0389 (8)	0.0022 (8)	0.0157 (7)	0.0065 (8)
C4	0.0406 (9)	0.0426 (10)	0.0291 (7)	−0.0039 (8)	0.0081 (6)	−0.0013 (8)
C5	0.0358 (8)	0.0445 (11)	0.0302 (7)	0.0046 (8)	0.0108 (6)	0.0025 (8)
C6	0.0299 (7)	0.0418 (10)	0.0267 (7)	0.0050 (7)	0.0059 (6)	0.0018 (7)
C7	0.0424 (9)	0.0309 (9)	0.0280 (7)	−0.0008 (8)	0.0104 (6)	−0.0002 (7)
C8	0.0569 (11)	0.0593 (14)	0.0278 (8)	0.0018 (10)	0.0043 (7)	0.0017 (9)
C9	0.0350 (8)	0.0384 (10)	0.0297 (7)	0.0113 (8)	0.0043 (6)	0.0045 (8)
C10	0.0354 (8)	0.0479 (12)	0.0376 (8)	0.0073 (8)	0.0037 (7)	0.0014 (9)
C11	0.0411 (10)	0.0771 (17)	0.0569 (12)	−0.0041 (11)	0.0015 (9)	−0.0111 (12)
C12	0.0513 (12)	0.121 (2)	0.0518 (12)	0.0018 (15)	−0.0036 (10)	−0.0304 (15)
C13	0.0527 (12)	0.113 (2)	0.0341 (10)	0.0121 (14)	0.0031 (9)	−0.0081 (12)
C14	0.0440 (9)	0.0632 (14)	0.0332 (9)	0.0088 (9)	0.0047 (7)	0.0068 (9)
C15	0.0833 (16)	0.0416 (12)	0.0662 (14)	−0.0151 (12)	0.0334 (12)	−0.0112 (11)
N16	0.0512 (9)	0.0489 (10)	0.0408 (8)	−0.0070 (8)	0.0159 (7)	0.0031 (8)
C17	0.0455 (11)	0.0840 (18)	0.0611 (12)	0.0212 (12)	0.0255 (10)	0.0193 (13)
C18	0.0403 (8)	0.0427 (10)	0.0332 (8)	0.0137 (9)	0.0121 (7)	0.0077 (8)
C19	0.0533 (11)	0.0663 (15)	0.0452 (10)	0.0238 (11)	0.0137 (9)	0.0159 (11)
C20	0.0852 (19)	0.0641 (17)	0.0700 (16)	0.0433 (16)	0.0252 (14)	0.0256 (14)
C21	0.100 (2)	0.0420 (14)	0.095 (2)	0.0247 (15)	0.0457 (18)	0.0173 (15)
C22	0.0753 (15)	0.0421 (13)	0.0755 (15)	0.0036 (12)	0.0277 (13)	−0.0037 (12)
C23	0.0492 (10)	0.0376 (11)	0.0473 (10)	0.0089 (9)	0.0137 (8)	0.0043 (9)

### Geometric parameters (Å, °)

**Table:**

Cl1—C8	1.772 (2)	C9—C14	1.402 (2)
Cl2—C10	1.7383 (18)	C10—C11	1.377 (3)
Cl3—C19	1.738 (3)	C11—C12	1.376 (3)
O1—C7	1.224 (2)	C11—H11	0.9300
O2—N16	1.408 (2)	C12—C13	1.355 (4)
O2—H2A	0.8200	C12—H12	0.9300
N1—C7	1.360 (2)	C13—C14	1.388 (3)
N1—C2	1.484 (2)	C13—H13	0.9300
N1—C6	1.496 (2)	C14—H14	0.9300
C2—C9	1.527 (3)	C15—H15A	0.9600
C2—C3	1.537 (2)	C15—H15B	0.9600
C2—H2	0.9800	C15—H15C	0.9600
C3—C4	1.508 (2)	C17—H17A	0.9600
C3—C15	1.529 (3)	C17—H17B	0.9600
C3—H3	0.9800	C17—H17C	0.9600
C4—N16	1.269 (2)	C18—C23	1.388 (3)
C4—C5	1.510 (3)	C18—C19	1.394 (3)
C5—C17	1.527 (3)	C19—C20	1.385 (4)
C5—C6	1.543 (2)	C20—C21	1.367 (4)
C5—H5	0.9800	C20—H20	0.9300
C6—C18	1.521 (3)	C21—C22	1.377 (4)
C6—H6	0.9800	C21—H21	0.9300
C7—C8	1.513 (3)	C22—C23	1.384 (3)
C8—H8A	0.9700	C22—H22	0.9300
C8—H8B	0.9700	C23—H23	0.9300
C9—C10	1.388 (3)		
			
N16—O2—H2A	109.5	C9—C10—Cl2	120.52 (14)
C7—N1—C2	117.79 (13)	C12—C11—C10	118.9 (2)
C7—N1—C6	120.37 (13)	C12—C11—H11	120.5
C2—N1—C6	119.18 (12)	C10—C11—H11	120.5
N1—C2—C9	114.70 (14)	C13—C12—C11	120.0 (2)
N1—C2—C3	107.79 (14)	C13—C12—H12	120.0
C9—C2—C3	114.42 (14)	C11—C12—H12	120.0
N1—C2—H2	106.4	C12—C13—C14	120.8 (2)
C9—C2—H2	106.4	C12—C13—H13	119.6
C3—C2—H2	106.4	C14—C13—H13	119.6
C4—C3—C15	110.84 (17)	C13—C14—C9	121.3 (2)
C4—C3—C2	110.21 (15)	C13—C14—H14	119.3
C15—C3—C2	111.38 (15)	C9—C14—H14	119.3
C4—C3—H3	108.1	C3—C15—H15A	109.5
C15—C3—H3	108.1	C3—C15—H15B	109.5
C2—C3—H3	108.1	H15A—C15—H15B	109.5
N16—C4—C3	125.40 (18)	C3—C15—H15C	109.5
N16—C4—C5	115.98 (16)	H15A—C15—H15C	109.5
C3—C4—C5	118.42 (14)	H15B—C15—H15C	109.5
C4—C5—C17	113.10 (16)	C4—N16—O2	112.12 (16)
C4—C5—C6	112.98 (14)	C5—C17—H17A	109.5
C17—C5—C6	109.75 (15)	C5—C17—H17B	109.5
C4—C5—H5	106.9	H17A—C17—H17B	109.5
C17—C5—H5	106.9	C5—C17—H17C	109.5
C6—C5—H5	106.9	H17A—C17—H17C	109.5
N1—C6—C18	111.03 (13)	H17B—C17—H17C	109.5
N1—C6—C5	111.49 (13)	C23—C18—C19	117.14 (19)
C18—C6—C5	109.74 (14)	C23—C18—C6	120.39 (16)
N1—C6—H6	108.2	C19—C18—C6	122.42 (19)
C18—C6—H6	108.2	C20—C19—C18	121.4 (2)
C5—C6—H6	108.2	C20—C19—Cl3	117.39 (19)
O1—C7—N1	122.74 (16)	C18—C19—Cl3	121.19 (18)
O1—C7—C8	117.68 (15)	C21—C20—C19	119.8 (2)
N1—C7—C8	119.57 (15)	C21—C20—H20	120.1
C7—C8—Cl1	108.57 (15)	C19—C20—H20	120.1
C7—C8—H8A	110.0	C20—C21—C22	120.5 (2)
Cl1—C8—H8A	110.0	C20—C21—H21	119.8
C7—C8—H8B	110.0	C22—C21—H21	119.8
Cl1—C8—H8B	110.0	C21—C22—C23	119.4 (3)
H8A—C8—H8B	108.4	C21—C22—H22	120.3
C10—C9—C14	115.36 (17)	C23—C22—H22	120.3
C10—C9—C2	122.45 (14)	C22—C23—C18	121.7 (2)
C14—C9—C2	122.10 (17)	C22—C23—H23	119.1
C11—C10—C9	123.57 (17)	C18—C23—H23	119.1
C11—C10—Cl2	115.90 (15)		
			
C7—N1—C2—C9	−121.63 (16)	C3—C2—C9—C10	−170.16 (16)
C6—N1—C2—C9	76.79 (18)	N1—C2—C9—C14	−119.16 (18)
C7—N1—C2—C3	109.65 (16)	C3—C2—C9—C14	6.2 (2)
C6—N1—C2—C3	−51.92 (19)	C14—C9—C10—C11	1.6 (3)
N1—C2—C3—C4	58.14 (18)	C2—C9—C10—C11	178.1 (2)
C9—C2—C3—C4	−70.73 (19)	C14—C9—C10—Cl2	−178.56 (15)
N1—C2—C3—C15	−65.3 (2)	C2—C9—C10—Cl2	−2.0 (2)
C9—C2—C3—C15	165.81 (17)	C9—C10—C11—C12	−1.1 (4)
C15—C3—C4—N16	−77.9 (2)	Cl2—C10—C11—C12	179.0 (2)
C2—C3—C4—N16	158.32 (18)	C10—C11—C12—C13	0.0 (4)
C15—C3—C4—C5	107.4 (2)	C11—C12—C13—C14	0.6 (4)
C2—C3—C4—C5	−16.4 (2)	C12—C13—C14—C9	−0.1 (4)
N16—C4—C5—C17	24.7 (2)	C10—C9—C14—C13	−1.0 (3)
C3—C4—C5—C17	−160.10 (18)	C2—C9—C14—C13	−177.52 (19)
N16—C4—C5—C6	150.15 (16)	C3—C4—N16—O2	−0.2 (3)
C3—C4—C5—C6	−34.6 (2)	C5—C4—N16—O2	174.59 (16)
C7—N1—C6—C18	77.29 (19)	N1—C6—C18—C23	52.7 (2)
C2—N1—C6—C18	−121.62 (16)	C5—C6—C18—C23	−71.05 (19)
C7—N1—C6—C5	−160.01 (15)	N1—C6—C18—C19	−129.93 (16)
C2—N1—C6—C5	1.1 (2)	C5—C6—C18—C19	106.36 (18)
C4—C5—C6—N1	42.4 (2)	C23—C18—C19—C20	−2.6 (3)
C17—C5—C6—N1	169.59 (18)	C6—C18—C19—C20	179.90 (18)
C4—C5—C6—C18	165.79 (15)	C23—C18—C19—Cl3	177.34 (14)
C17—C5—C6—C18	−67.0 (2)	C6—C18—C19—Cl3	−0.2 (2)
C2—N1—C7—O1	12.7 (2)	C18—C19—C20—C21	1.9 (3)
C6—N1—C7—O1	174.07 (17)	Cl3—C19—C20—C21	−178.02 (19)
C2—N1—C7—C8	−166.86 (16)	C19—C20—C21—C22	0.2 (4)
C6—N1—C7—C8	−5.5 (2)	C20—C21—C22—C23	−1.5 (4)
O1—C7—C8—Cl1	−88.46 (18)	C21—C22—C23—C18	0.7 (3)
N1—C7—C8—Cl1	91.14 (18)	C19—C18—C23—C22	1.3 (3)
N1—C2—C9—C10	64.5 (2)	C6—C18—C23—C22	178.83 (17)

### Hydrogen-bond geometry (Å, °)

**Table:**

*D*—H···*A*	*D*—H	H···*A*	*D*···*A*	*D*—H···*A*
O2—H2A···O1^i^	0.82	2.01	2.7515 (18)	150
C12—H12···Cl2^ii^	0.93	2.68	3.485 (3)	145
C20—H20···O1^iii^	0.93	2.42	3.158 (3)	136

Symmetry codes: (i) *x*+1/2, −*y*+1/2, *z*−1/2; (ii) *x*, −*y*, *z*−1/2; (iii) *x*+1/2, *y*−1/2, *z*.
